# A Patient-Centered Approach to Guide Follow-Up and Adjunctive Testing and Treatment after First Rib Resection for Venous Thoracic Outlet Syndrome Is Safe and Effective

**DOI:** 10.3390/diagnostics8010004

**Published:** 2018-01-23

**Authors:** Colin P. Ryan, Nicolas J. Mouawad, Patrick S. Vaccaro, Michael R. Go

**Affiliations:** 1 Division of Vascular Diseases and Surgery, The Ohio State University College of Medicine, Columbus, OH 43210, USA; cryan7209@gmail.com (C.P.R.); patrick.vaccaro@osumc.edu (P.S.V.); 2 McClaren Bay Heart and Vascular, McClaren Regional Medical Center, Flint, MI 48532, USA; nicolas.mouawad@osumc.edu

**Keywords:** venous thoracic outlet syndrome, thoracic outlet syndrome, first rib resection, thoracic outlet decompression, thrombolysis, Paget-Schroetter syndrome, McCleery syndrome, deep venous thrombosis

## Abstract

Controversies in the treatment of venous thoracic outlet syndrome (VTOS) have been discussed for decades, but still persist. Calls for more objective reporting standards have pushed practice towards comprehensive venous evaluations and interventions after first rib resection (FRR) for all patients. In our practice, we have relied on patient-centered, patient-reported outcomes to guide adjunctive treatment and measure success. Thus, we sought to investigate the use of thrombolysis versus anticoagulation alone, timing of FRR following thrombolysis, post-FRR venous intervention, and FRR for McCleery syndrome (MCS) and their impact on patient symptoms and return to function. All patients undergoing FRR for VTOS at our institution from 4 April 2000 through 31 December 2013 were reviewed. Demographics, symptoms, diagnostic and treatment details, and outcomes were collected. Per “Reporting Standards of the Society for Vascular Surgery for Thoracic Outlet Syndrome”, symptoms were described as swelling/discoloration/heaviness, collaterals, concomitant neurogenic symptoms, and functional impairment. Patient-reported response to treatment was defined as complete (no residual symptoms and return to function), partial (any residual symptoms present but no functional impairment), temporary (initial improvement but subsequent recurrence of any symptoms or functional impairment), or none (persistent symptoms or functional impairment). Sixty FRR were performed on 59 patients. 54.2% were female with a mean age of 34.3 years. Swelling/discoloration/heaviness was present in all but one patient, deep vein thrombosis in 80%, and visible collaterals in 41.7%. Four patients had pulmonary embolus while 65% had concomitant neurogenic symptoms. In addition, 74.6% of patients were anticoagulated and 44.1% also underwent thrombolysis prior to FRR. Complete or partial response occurred in 93.4%. Of the four patients with temporary or no response, further diagnostics revealed residual venous disease in two and occult alternative diagnoses in two. Use of thrombolysis was not related to FRR outcomes (*p* = 0.600). Performance of FRR less than or greater than six weeks after the initiation of anticoagulation or treatment with thrombolysis was not related to FRR outcomes (*p* = 1). Whether patients had DVT or MCS was not related to FRR outcomes (*p* = 1). No patient had recurrent DVT. From a patient-centered, patient-reported standpoint, VTOS is equally effectively treated with FRR regardless of preoperative thrombolysis or timing of surgery after thrombolysis. A conservative approach to venous interrogation and intervention after FRR is safe and effective for symptom control and return to function. Additionally, patients with MCS are effectively treated with FRR.

## 1. Introduction

Controversies in the treatment of venous thoracic outlet syndrome (VTOS) include the use of preoperative thrombolysis, timing of first rib resection (FRR) after presentation, surgical approach, and the role of post-FRR venous intervention. The lack of true comparative analysis data in thoracic outlet syndrome in general, and VTOS in particular, has resulted in calls for objective reporting criteria and an emphasis on diagnostics to measure treatment success [1,2,3].

While this clearly fills an important and well-recognized gap in the VTOS literature, in our practice we have historically focused on patient-centered, patient-reported measures of treatment success, and thus do not pursue duplex or venography after FRR, and do not treat incidentally identified lesions, unless the patient has significant residual symptoms or functional impairment. In this setting, our patient population is suited to address some of the existing controversies in the treatment of VTOS, especially as they relate to patient-reported outcomes and conservative management of residual venous disease after FRR.

Thus, in a practice emphasizing patient-reported outcomes over venous patency, we sought to describe the effect of pre-operative thrombolysis, timing of FRR, and a conservative approach to post-operative vein management on the results of treatment of VTOS. Additionally, we investigated the role of FRR in the treatment of symptoms and functional impairment from McCleery syndrome (MCS).

## 2. Materials and Methods

This study was approved by the Ohio State University Institutional Review Board (Protocol 2013H0067 approved 2/22/13 with ongoing approval). A retrospective review analyzing all patients undergoing FRR for VTOS from 4 April 2000 through 31 December 2013 at our institution was performed. VTOS was defined according to the “Reporting Standards of the Society for Vascular Surgery for Thoracic Outlet Syndrome” as a presence of consistent history, consistent examination, and imaging demonstrating DVT or venous abnormality at the thoracic outlet, with the exception that asymptomatic incidentally found venous compression without DVT was not treated [3]. No patients had concomitant arterial TOS, but some did have neurogenic symptoms.

Also per “Reporting Standards of the Society for Vascular Surgery for Thoracic Outlet Syndrome”, symptoms were described as swelling/discoloration/heaviness, collaterals, concomitant neurogenic symptoms, and functional impairment; this data was collected for all patients in our practice, including those treated prior to the publication of the reporting standards.

Patients presenting with VTOS and DVT all were anticoagulated for a total of three months from presentation. Patients underwent additional pre-operative thrombolysis based on the judgment of the treating surgeon. If thrombolysis occurred, it was performed using ultrasound-accelerated thrombolysis (EKOS Corporation, Bothell, WA, USA) while infusing 1 mg/h tPA for 12–24 h. Venoplasty for residual stenosis after thrombolysis was performed using low pressure, smaller diameter balloons only as a temporizing measure, and stents were avoided. The decision of when to perform FRR after anticoagulation and thrombolysis was made by the treating surgeon, and all FRR were performed via the transaxillary approach with anterior scalenotomy and external venolysis but without venous reconstruction.

Response to treatment included outcomes defined in “Reporting Standards of the Society for Vascular Surgery for Thoracic Outlet Syndrome”, including overall subjective status, return to function, objective examination, and—most importantly—patient-reported symptoms. Response was further defined as complete (no residual symptoms and return to function), partial (any residual symptoms present but no functional impairment), temporary (initial improvement but subsequent recurrence of any symptoms or functional impairment), or none (persistent symptoms or functional impairment), similar to the methodology of Derkash et al. and subsequently utilized by Degeorges et al. [4,5]. Outcomes were further categorized as favorable or unfavorable, as done by Orlando et al. [6]. Patients were considered to have complete response only if absolutely no swelling, discoloration, heaviness, collaterals, or any upper extremity symptoms of any kind were reported or identified on exam.

Follow-up was heavily influenced by patient-reported symptoms and functional outcomes rather than anatomic or patency measures. All patients returned at one month for a history and examination. Those with no residual symptoms had no further follow-up or assessments arranged, but were told to return if any symptoms recurred. In any patient with residual symptoms or functional limitation, further venous interrogation and intervention were undertaken.

Data were stored in a password-protected database on a secured computer. Deidentified data were imported to SPSS (Version 22, IBM Corporation, Armonk, NY, USA) for statistical analysis. Summaries of the entire cohort were created using medians and ranges for continuous measures and frequencies and percentages for categorical measures. Chi-squared analysis of outcomes measures for variables with expected counts greater than five was performed. Fisher exact testing was utilized for variables with expected counts less than five.

## 3. Results

Sixty FRR in 59 patients were performed for VTOS. DVT was diagnosed in 48 patients (80%), while MCS was diagnosed in 12 (20%).

Table 1 shows summaries of the demographic and presenting features of the VTOS cohort. Favorable outcome indicates complete or partial response. Patients presenting with venous distention were more likely to have an unfavorable outcome.

Twenty-seven patients were female with a mean age of 34.3 years. Eight patients (13.6%) had a family history of coagulopathy and one patient had a parent with VTOS. Thirty-one percent of patients were unemployed or disabled, 28.8% were athletes, 16.9% were sedentary workers, and 16.9% were repetitive overhead workers.

Swelling was present in 98.3% (97.9% of patients with DVT, 100% of patients with MCS). Venous distention was present in 41.7% (35.4% of patients with DVT, 58.3% of patients with MCS). Four patients had pulmonary embolus at presentation. Sixty-five percent had concurrent upper extremity neurologic symptoms.

**Figure 1:**
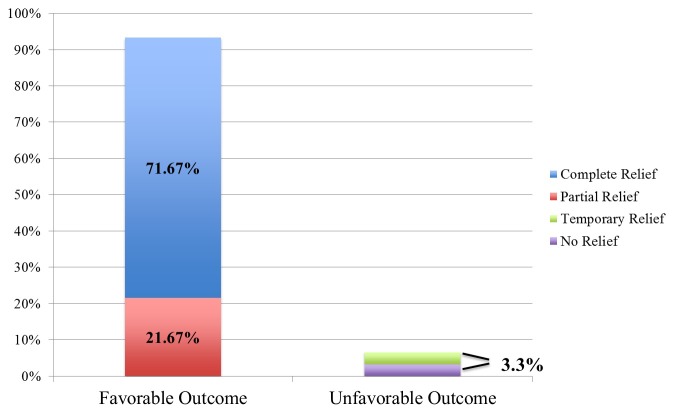
Response following first rib resection (FRR).

Regarding treatment, 78% of patients were anticoagulated (95.8% of patients with DVT, 0% of patients with MCS). In addition, 54.1% of patients with DVT underwent thrombolysis prior to FRR. Overall, complete response was achieved in 71.7% of patients, partial response was achieved in 21.7%, and temporary or no response each occurred in 3.3% (Figure 1).

**Figure 2:**
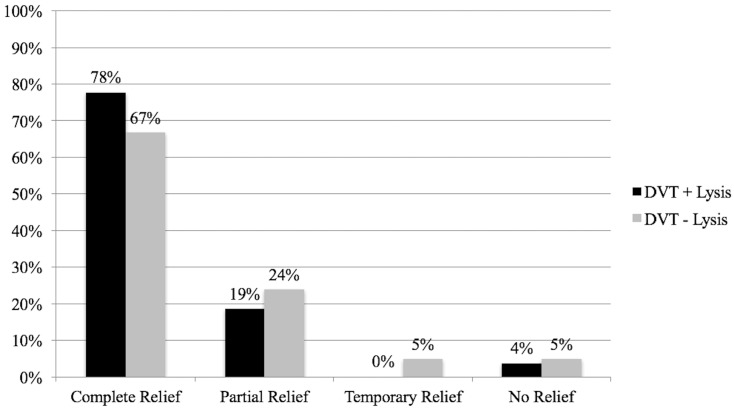
Outcomes with or without preoperative thrombolysis following FRR.

Performance of preoperative thrombolysis was not related to FRR outcomes (*p* = 0.620) as demonstrated in Figure 2.

**Figure 3:**
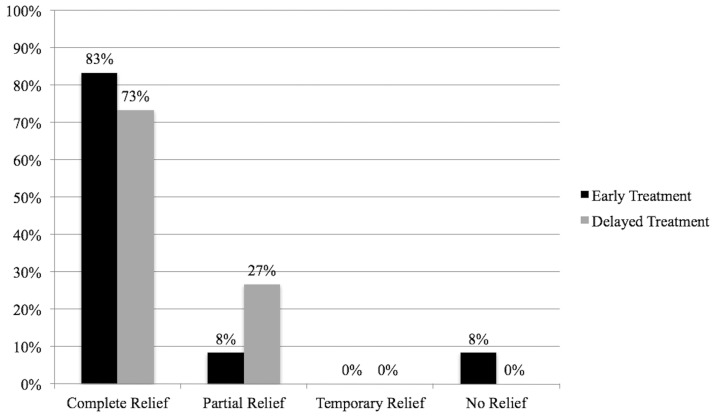
Outcomes after FRR less than or greater than six weeks after presentation.

Performance of FRR less than or greater than six weeks after preoperative thrombolysis was not related to FRR outcomes (*p* = 0.444; Figure 3).

Whether patients were diagnosed with a DVT or had MCS only was not related to FRR outcomes (*p* = 0.796). No patient had a recurrent DVT. Pneumothorax occurred in 10%. By definition, of the 43 patients who had complete responses, all were able to return to their preoperative occupation or athletic activity and had no residual symptoms. Of the 13 patients who had partial responses, all had mild residual arm swelling but had significant symptomatic improvement and were able to return to their pre-operative occupational or athletic function. Median follow-up was 3.7 months.

By VTOS subclass, 72% of DVT patients and 67% of MCS patients achieved a complete response. Overall, of those with temporary or no response, two patients had chronic axillary vein occlusion after FRR and underwent subsequent venoplasty; one achieved complete response after this while the other demonstrated re-thrombosis and return of symptoms treated with ongoing anticoagulation but does not have functional limitation. The other two patients with temporary or no response had patent axillosubclavian veins on interrogation, but one was diagnosed with chronic regional pain syndrome and the other was found to have severe cervical degenerative disc disease with median and ulnar nerve entrapment.

## 4. Discussion

In this cohort of VTOS patients, an analysis emphasizing patient-centered and functional outcomes demonstrated that FRR is effective for the treatment of symptoms of VTOS including swelling/discoloration/heaviness, collaterals, and functional impairment. Furthermore, no differences in these outcomes were identified based on use of preoperative thrombolysis or the timing of FRR after thrombolysis in VTOS. Lastly, FRR was found to be effective in treating symptoms associated with MCS.

Historically, outcomes for VTOS have focused on DVT recurrence and axillosubclavian vein patency. In our practice, we have emphasized patient-centered and functional outcomes, as available evidence suggests that most VTOS patients will do well from a DVT recurrence standpoint after FRR, even if venous stenosis or occlusion persists [7]. We did not have any DVT recurrence in this series. Furthermore, Machleder, in his 1993 report, recognized that significant numbers of patients left with chronically occluded veins after FRR would still go on to have excellent symptom relief and functional recovery [7]. Heron et al. in 1991 showed that in 54 patients with spontaneous axillosubclavian thrombosis treated with anticoagulation alone, though 22% of patients had persistent venous occlusion, venous patency did not correlate with symptom resolution [8]. In our practice, we similarly have found that a majority of patients do well symptomatically, often even in the setting of chronically occluded veins, and so patient-reported symptoms, functional restoration, and satisfaction with treatment form the basis for our recommendations after FRR. Thus, we do not routinely interrogate the axillosubclavian vein for patency, though we do so aggressively if the patient is anything other than totally asymptomatic. Our outcomes with this approach to the disease, in addition to how thrombolysis and timing of surgery may affect them, formed the basis for this study.

The timing of FRR for VTOS in patients with DVT who undergo preoperative thrombolysis remains controversial. Machleder initially advocated for delaying surgery as long as three months after thrombolysis to minimize complication risk caused by acute inflammation from DVT and to avoid the thrombogenicity of a recently thrombophlebitic vein [7]. Advocates of thrombolysis followed by immediate surgery remain concerned for rethrombosis prior to FRR [9,10]. Recently, Elixène et al. found that, of patients treated with thrombolysis followed by FRR within 10 days, 100% demonstrated complete resolution of symptoms at a median follow-up of 240 months [11]. Angle et al. similarly reported that early rib resection after thrombolysis is safe and may reduce the risk of rethrombosis during a longer waiting interval [9]. Our data showed that the time to FRR of less than or greater than six weeks after thrombolysis did not statistically significantly impact symptomatic or functional outcomes. Ninety-one percent of patients undergoing FRR within six weeks of thrombolysis achieved symptom resolution, and 100% of those undergoing FRR six weeks or more after thrombolysis achieved symptom resolution. This finding stands in agreement with de León et al., who reported complete remission of symptoms in 95.5% of patients following FRR regardless of the timing of surgery [12].

Alternatively, some advocate anticoagulation only prior to FRR for VTOS, and do not believe that routine preoperative thrombolysis of DVT is necessary. Guzzo et al. compared patients who had thrombolysis prior to FRR with those who had anticoagulation only prior to FRR in a series of 110 procedures [13]. In both cohorts, 91% of patients had symptomatic improvement and venous patency at one year [13]. This finding was substantiated by Sabeti et al., who reported that patients who underwent thrombolysis had a higher rate of venous patency but a similar rate of symptom resolution compared to those who were treated with anticoagulation alone [14]. Despite these findings, anticoagulation with thrombolysis continues to be a more common practice.

In our study, functional outcomes were similar after FRR for both DVT and MCS patients. A number of studies examining FRR for VTOS have included MCS patients without performing separate analyses, and few studies have examined outcomes for these patients specifically. Likes et al. presented a cohort of 19 patients who underwent 20 FRR for MCS. At the date of last follow-up, all patients were symptom-free following surgical intervention [15]. In a subgroup analysis, de León et al. similarly reported an 81% complete symptom resolution rate after FRR for MCS (nine of 11 patients) [12].

Lastly, transaxillary, infraclavicular, and paraclavicular surgical approaches to FRR for VTOS have all been advocated [6,16]. A paraclavicular approach to FRR clearly affords the best exposure of the vein, and this technique makes sense when open venous reconstruction is planned [16]. In our practice, however, we typically perform transaxillary resections for VTOS with the understanding that we will rely on transcatheter venous procedures post-operatively in the minority of patients who do not achieve symptom resolution or functional recovery. We have been able to achieve satisfactory patient-reported outcomes while minimizing post-operative venous interrogation and intervention, and thus have not found a compelling need for aggressive intra-operative venous reconstruction afforded by a paraclavicular approach.

Significant limitations of this study are apparent. This is a retrospective study without randomization, thus selection bias affected the choices of pre-operative thrombolysis and timing of FRR. Because follow-up was guided completely by patient-reported symptoms, there was no consistent long-term follow-up for all patients. Although all patients who achieved satisfactory symptom control were instructed to call with recurrent problems, it is clearly possible that some did not. Thus, while the short 3.7 months follow-up may reflect that many patients did well without residual symptoms or functional limitation and did not seek further care, it may also falsely increase our favorable outcome rate if patients with recurrence chose not to initiate further care. And because our approach did not include universal venous interrogation after FRR, we do not have complete data on post-operative patency or how that may have affected patient-centered outcomes. Finally, as attention to patient-centered outcomes has increased, the availability of instruments to quantify outcomes for diseases such as TOS that manifest with relatively subjective symptoms has increased, and we plan to implement these instruments in future studies of our approach to VTOS.

## 5. Conclusions

A patient-centered approach to guiding post-FRR testing and intervention is safe and reasonable. Symptom control and return to function are effectively achieved with FRR regardless of preoperative thrombolysis or the timing of surgery after thrombolysis. Additionally, patients with MCS can achieve very good symptom relief after FRR.

## Figures and Tables

**Table 1:** Association of patient presentation variables with surgical outcomes.

Factor	Number (%)	% Favorable Outcome	*p*-Value
Gender			
Male	28 (45.8%)	92.8%	1
Female	32 (54.2%)	93.8%	1
Smoking Status			
Nonsmoker	45 (70.3%)	95.6%	-
Past/Current Smoker	15 (29.7%)	86.7%	0.258
Occupation			
Unemployed/Disabled/Retired	18 (30.5%)	88.8%	0.572
Student/Sedentary Laborer	14 (23.3%)	92.4%	1
Student Athlete	17 (28.3%)	91.4%	1
Overhead/Physical Laborer	10 (16.7%)	90%	1
Presentation			
Upper Extremity Swelling	59 (98.3%)	-	1
Venous Distension	25 (41.7%)	84.0%	0.026
Deep Venous Thrombosis	48 (80%)	94.0%	0.528
Pulmonary Embolus	4 (6.7%)	100%	1
Neurologic Symptoms	39 (65%)	92.3%	1
Family Clotting History	9 (15%)	100%	1
